# Organizational factors influencing recreational therapy program effectiveness in veterans’ nursing homes: A mixed-methods approach

**DOI:** 10.1371/journal.pone.0353395

**Published:** 2026-07-16

**Authors:** Mohammad Najeh Samara, Kimberly D. Harry, Brittany Weissman, Kara V. Dopp

**Affiliations:** 1 School of Systems Science and Industrial Engineering, Binghamton University, Binghamton, New York, United States of America; 2 New York State Veterans Home at Oxford, Oxford, New York, United States of America; Noida International University, INDIA

## Abstract

**Background:**

Veterans’ nursing homes face significant challenges in delivering effective recreational therapy (RT) programs due to complex organizational factors and unique veteran care needs. Limited research has systematically examined organizational determinants from leadership perspectives, despite leaders’ critical roles in resource allocation and program implementation.

**Objective:**

This study examined organizational factors influencing recreational therapy program effectiveness in a veterans’ nursing home through comprehensive analysis of leadership perspectives using a mixed-methods approach.

**Methods:**

A cross-sectional survey was administered to 19 leadership personnel at NYSVETS Home @ Oxford. The 28-question survey assessed RT program effectiveness across five well-being domains, organizational strengths and weaknesses, participation barriers, and leadership involvement patterns. Analysis included descriptive statistics, correlation analysis, and systematic thematic coding of 192 qualitative responses.

**Results:**

Leadership rated overall RT program effectiveness highly (78.9%, 95% CI: 60.6–97.3%), with Physical well-being showing the highest impact (3.75 ± 0.62, 95% CI: 3.36–4.14) and no significant differences across domains (χ² = 0.563, p = 0.967, Kendall’s W = 0.007). Organizational strengths significantly outweighed weaknesses (U = 24.0, p = 0.010, effect size r = 0.741), with activity variety (89.5%) as the primary asset and resource limitations (52.6%) as the key deficit. Strong positive correlations emerged between engaged veterans and dedicated staff (r = 0.782, 95% CI: [0.508, 0.912]). Leadership demonstrated strong mission-vision alignment (76.5%) but involvement intensity showed no significant association with effectiveness (r = 0.335, 95% CI: [−0.214, 0.723], p = 0.223). A Resource Adequacy Index of 29.7% indicated critical capacity limitations. Qualitative analysis validated quantitative findings, with Social Interaction (40 mentions) and Physical Health (36 mentions) themes supporting effectiveness priorities while revealing veteran-specific challenges including generational preferences for individualized programming.

**Conclusions:**

This study provides comprehensive evidence for organizational factors influencing RT effectiveness, demonstrating both program strengths and critical improvement areas. The organizational priority matrix and resource allocation analysis provide evidence-based tools for strategic planning and quality improvement initiatives, informing policy development and practical guidance for healthcare administrators.

## Introduction

### Background

Recreational therapy (RT) programs in veterans’ nursing homes serve a critical role in promoting multi-dimensional well-being for aging military populations [1]. These programs encompass a wide variety of activities, such as physical exercise, creative arts, group outings, music therapy, cognitive games, and spiritual services, which are designed to support the diverse needs and preferences of veteran residents [2–5]. However, the effectiveness of RT in these settings is shaped by a constellation of organizational factors, including leadership support, resource allocation, staffing adequacy, and the alignment of program design with the unique therapeutic needs of veteran residents [6–8].

Leadership perspectives represent a particularly important yet understudied aspect of RT program effectiveness. Engaged leadership is vital for sustaining and enhancing therapeutic programs, as leaders are responsible for providing resources, fostering open communication, and supporting staff [8]. Effective leaders model accessibility and responsiveness, contributing to a positive safety climate and stronger program outcomes in nursing homes [7], while leadership commitment to resident-centered care helps mitigate challenges related to staff morale and organizational culture [7]. Beyond leadership, staffing constraints represent one of the most persistent organizational challenges, as difficulties in recruiting and retaining qualified personnel hinder program implementation and limit the scope of care provided to veterans [6,8]. While creative staffing solutions and flexible scheduling can provide some relief [9], the interdisciplinary nature of RT programs necessitates robust training and cross-disciplinary engagement [10]. Similarly, resource limitations including funding, physical space, and equipment further restrict program reach and effectiveness [6,8].

These organizational challenges are further complicated by the distinctive characteristics and needs of the veteran population. Many veterans require trauma-informed care, which is based on principles of safety, trust, collaboration, empowerment, and choice [11,12]. However, implementation of trauma-informed approaches is often challenged by limited regulatory guidance and the need for significant cultural change within organizations [12,13]. Additionally, generational differences among veterans lead to diverse therapeutic priorities, with older veterans often emphasizing physical rehabilitation and younger veterans prioritizing mental health support [14]. The intersection of organizational constraints and veteran-specific needs manifests in multiple participation barriers including health and mobility limitations, environmental constraints, and motivational challenges [15,16]. Chronic illness and restricted mobility can diminish veterans’ ability to engage in RT activities, while inadequate facilities and accessibility issues may further discourage participation. Motivation, often undermined by mental health concerns or insufficient encouragement, represents an additional barrier [17,18]. In contrast, facilitators like social support, tailored program offerings, and a supportive organizational culture can meaningfully enhance participation when present [15,19,20].

### Objectives and contribution

Despite the recognized importance of these organizational factors, their influence on RT program effectiveness from the perspective of leadership remains inadequately explored. Most previous research has prioritized clinical outcomes, leaving a significant gap in understanding how organizational determinants and veteran-specific needs interact to shape program success. Addressing this complexity requires mixed-methods research capable of integrating quantitative assessments and qualitative insights to produce a more holistic understanding. Therefore, this study seeks to address these critical gaps by systematically examining organizational factors that influence recreational therapy program effectiveness in a veterans’ nursing home, with a specific focus on leadership perspectives. Through a mixed-methods approach, we aim to first identify critical organizational determinants, then analyze their relationships with program effectiveness across well-being domains, and subsequently generate evidence-based recommendations for quality improvement and strategic planning in long-term care settings for veterans.

## Methods

### Study design and Setting

This study employed a mixed-methods, cross-sectional survey design to assess organizational factors influencing the effectiveness of Recreational Therapy (RT) programs in a veterans’ nursing home setting. The research was conducted at the New York State Veterans’ Home at Oxford (NYSVETS Home). The facility operates an established RT program that transitioned from a traditional activities program within the past decade.

### Participants and Data Collection

Eligible participants included organizational leadership personnel, therapy staff, support personnel, and administrators affiliated with the RT program. Recruitment targeted personnel involved in recreational therapy program delivery, coordination, oversight, and resident engagement across organizational roles at the facility, and participation was voluntary. The data collection period was from March 1 to June 9, 2025. A total of 19 valid survey responses were obtained. Data were collected via an online survey distributed through the Qualtrics platform, ensuring participant anonymity and confidentiality.

### Survey instrument

The survey instrument was developed to capture quantitative and qualitative assessments of RT program effectiveness, organizational strengths and weaknesses, participation barriers, leadership involvement, and program alignment with the facility’s mission. The instrument included:

- Closed-ended questions using Likert-type and multiple-selection formats.
- Domain-specific questions assessing physical, cognitive, emotional, social, and spiritual well-being impacts.
- Open-ended questions to capture qualitative insights regarding program effectiveness, barriers, and growth opportunities.

Content validity was established through review by the research team, including subject matter experts in recreational therapy and healthcare systems engineering, to ensure alignment with the study’s theoretical framework and relevance to the long-term care context. Internal consistency of the five-domain effectiveness scale was assessed using Cronbach’s alpha, yielding α = 0.864, indicating excellent reliability [21]. The survey required approximately 10–15 minutes to complete. Formal pilot testing was not conducted prior to administration, which is acknowledged as a limitation; however, the content review process and strong internal consistency provide reasonable evidence of instrument quality for this exploratory study.

### Statistical analysis

#### Data preparation.

Data cleaning procedures included validation of response completeness, identification and removal of invalid responses, and verification of logical consistency across related questions. Missing data were handled using complete case analysis, whereby only participants with valid responses for each specific variable were included in the corresponding analysis, resulting in variable-specific sample sizes (experience data: n = 17; timeline data: n = 16; position data: n = 16; leadership involvement: n = 15). This approach was selected given the small overall sample size and the exploratory nature of the study, as imputation methods would require assumptions regarding missing data mechanisms that could not be reliably verified with the available sample size. Missing data patterns were examined descriptively to assess potential response bias. Differential completion rates across survey sections (59.6% to 100%) suggested that non-response was more common for organizationally sensitive questions, which may indicate potential non-random missingness within organizational factors items.

### Quantitative analysis

**Descriptive Statistics:** Frequencies, percentages, means, standard deviations, and 95% confidence intervals were calculated for all survey variables. Response completeness was assessed across survey sections.

**Comparative Analysis:** Chi-square goodness-of-fit tests were used to assess associations between categorical variables [22]. Prior to analysis, expected cell frequencies were evaluated for each test to verify assumptions. For analyses where expected cell frequencies met the minimum threshold of 5, standard chi-square inference was applied: the organizational factor category distribution analysis (expected cell frequency = 31.7) and the leadership role distribution analysis (expected cell frequency = 9.5) met this criterion. For analyses where expected cell frequencies fell below this threshold due to the small sample size, a Monte Carlo permutation test (10,000 simulations) was employed as an assumption-robust alternative [23]: the role category distribution analysis (expected = 4.0), the association between individual experience and program implementation timeline (expected ≈ 1.0), and the cross-tabulation of leadership experience with alignment perspectives (expected ≈ 1.06) all required this approach. Effect sizes are reported as Cramér’s V for all chi-square analyses. Friedman tests [24] compared effectiveness ratings across well-being domains. Mann-Whitney U tests compared response patterns between groups [25].

**Correlation Analysis:** Pearson correlation coefficients were calculated to examine relationships between organizational factors. Statistical significance was determined using t-tests with Bonferroni correction applied across all comparisons (adjusted α = 0.0014). Correlations with |r| > 0.3 were additionally retained for practical interpretation as indicators of meaningful effect size, following [26], and are reported descriptively in Table S1 regardless of statistical significance. Confidence intervals (95%) were calculated using Fisher’s z transformation.Click or tap here to enter text.

**Priority Analysis:** Organizational factors were ranked by mention frequency and categorized into priority levels using percentage-based thresholds derived from the sample size (n = 19). Factors endorsed by more than 50% of respondents (≥10 mentions) were classified as HIGH priority, reflecting majority consensus among organizational personnel. Factors endorsed by 25–49% of respondents (5–9 mentions) were classified as MEDIUM priority, and those endorsed by fewer than 25% of respondents (<5 mentions) as LOW priority. This stratification approach is consistent with frequency-based priority-setting frameworks used in healthcare quality improvement research [27] and ensures thresholds are anchored to the sample rather than applied arbitrarily.

### Qualitative analysis

Open-ended responses underwent systematic thematic analysis [28]. Coding credibility was established through a systematic multi-step process: an initial codebook was developed inductively from response content by the first author, applied consistently across all 192 responses, and iteratively refined through analytic memos documenting coding decisions at each stage. The coding framework was subsequently reviewed by the research team to ensure alignment with the study’s theoretical framework and research objectives, with any interpretive discrepancies resolved through discussion until consensus was reached. Thematic saturation was assessed iteratively during coding, with no new themes emerging in the final 20% of responses, supporting adequate saturation within the study context. Response content was coded into thematic categories, with mention frequencies calculated across themes. Qualitative themes were triangulated with quantitative findings to assess convergent validity and identify implementation insights not captured in structured questions [29].

### Mixed-methods integration

This study employed a convergent parallel mixed-methods design [30], in which quantitative and qualitative data were collected concurrently, analyzed independently, and then systematically integrated during interpretation. Integration was operationalized through two sequential steps. First, quantitative priority rankings of organizational factors were compared against qualitative theme prominence rankings to assess convergence or divergence. Second, quantitative effectiveness ratings for each well-being domain were mapped against corresponding qualitative theme frequencies, identifying domains where both data strands produced consistent findings (convergence) and domains where qualitative data provided explanatory depth beyond quantitative results (expansion). Integration quality was guided by the criteria of completeness, adherence, and rigor outlined by [31], ensuring that the mixed-methods approach generated insights not achievable through either strand alone.

### Statistical software

All analyses were conducted using Python version 3.10, with significance levels set at α = 0.05. Effect sizes (Cohen’s d, Cramér’s V) were calculated to assess practical significance beyond statistical significance.

### Ethical considerations

This study was conducted in compliance with ethical standards and was approved by the Binghamton University Institutional Review Board (IRB). Informed consent was obtained from all participants prior to survey initiation, ensuring that participants were fully aware of the study’s purpose, procedures, potential risks, and their rights as research subjects. Participation was entirely voluntary, and participants retained the right to withdraw from the study at any time without penalty or loss of benefits. To safeguard participant privacy, all survey responses were collected anonymously, and no personally identifiable information was linked to the data. Additionally, employers and organizational leadership were not provided with access to individual responses or participation records, ensuring confidentiality throughout the study. Data were securely stored using password-protected systems, and only authorized research personnel had access to the dataset. These measures were implemented to minimize any potential risk of breach of confidentiality and to protect the integrity of participant contributions.

## Results

### Participant characteristics and response patterns

Data cleaning procedures were applied to ensure analytical validity, including removal of one invalid response each for (experience) and (program timeline). The final analytical sample included 19 total valid responses, with variable-specific sample sizes of 17 participants for experience data, 16 participants for timeline data, and 16 participants for position data. Response rates for key variables exceeded 90%, with invalid responses systematically identified and excluded to maintain data integrity. Table 1 presents the demographic and professional characteristics of the study participants. The majority of participants (47.1%) had 1–5 years of experience with the RT program, while the program implementation timeline showed the greatest concentration (37.5%) in the 6–10-year range. Given that the 4x4 contingency table structure resulted in expected cell frequencies of approximately 1.0, well below the minimum threshold of 5, a Monte Carlo permutation test (10,000 simulations) was used to assess the association between individual experience and program implementation timeline, revealing no significant association (p = 0.631), indicating that participants’ tenure was independent of when the program was established. The organizational role distribution reflected the collaborative and operationally distributed nature of RT program implementation at the study site, with operational staff comprising the majority of respondents (68.8%), alongside senior leadership (12.5%), support staff (12.5%), and middle management (6.2%). All role categories included personnel directly involved in RT program delivery, coordination, oversight, or resident engagement. As expected cell frequencies (4.0) fell below the minimum threshold of 5, a Monte Carlo permutation test (10,000 simulations) was employed, confirming that the distribution differed significantly across organizational roles (p < 0.001, Cramér’s V = 0.741).

**Table 1:** Participant professional characteristics (N = 19).

Characteristic	n	%	95% CI
**Experience with RT Program** (n = 17)			
1-5 years	8	47.1	23.3-70.8
Less than 1 year	4	23.5	3.4-43.7
6-10 years	3	17.6	0.0-35.8
More than 10 years	2	11.8	0.0-27.1
**RT Program Implementation Timeline** (n = 16)			
6-10 years	6	37.5	13.8-61.2
1-5 years	4	25.0	3.8-46.2
Less than 1 year	3	18.8	0.0-37.9
More than 10 years	3	18.8	0.0-37.9
**Organizational Role Distribution** (n = 16)			
Operational Staff	11	68.8	45.2-92.4
Senior Leadership	2	12.5	0.0-28.7
Support Staff	2	12.5	0.0-28.7
Middle Management	1	6.2	0.0-18.1

*Note: CI = Confidence Interval*

**Fig 1:**
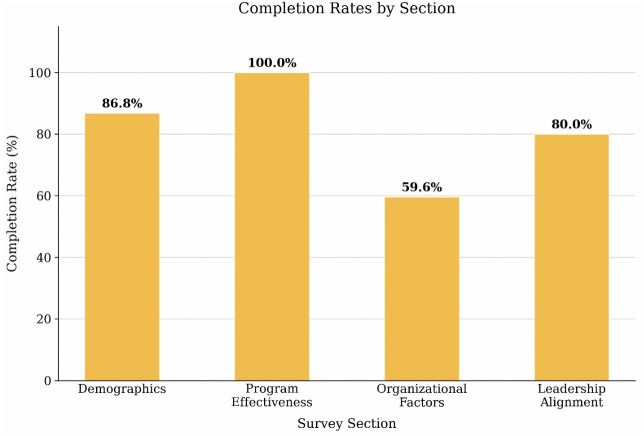
Response completion rates by survey section showing differential completion patterns across question domains.

Response completeness varied significantly across survey sections (Fig 1). Program Effectiveness questions achieved the highest completion rate (100%), followed by Demographics (86.8%) and Leadership Alignment (80.0%). Organizational Factors showed the lowest completion rate (59.6%), indicating greater respondent difficulty or reluctance to complete questions about organizational barriers and facilitators.

### Program effectiveness assessment

#### Overall program effectiveness.

Leadership perspectives on overall RT program effectiveness showed predominantly positive ratings (Table 2). The majority of respondents (78.9%) rated the program as highly effective, with 42.1% rating it as “very effective” and 36.8% as “effective.” Only 10.5% rated the program as merely “sufficient,” and 5.3% as “less effective.” No respondents rated the program as “ineffective.”

**Table 2:** Overall program effectiveness ratings.

Effectiveness Rating	n	%	95% CI
Very effective	8	42.1	19.9-64.3
Effective	7	36.8	15.2-58.5
Sufficient	2	10.5	0.0-24.3
Less effective	1	5.3	0.0-15.3
Ineffective	0	0.0	–
**Total**	**18**	**100.0**	

*High Effectiveness Rate (Very + Effective): (78.9%, 95% CI: 60.6–97.3%)*

### Domain-specific impact analysis

**Fig 2:**
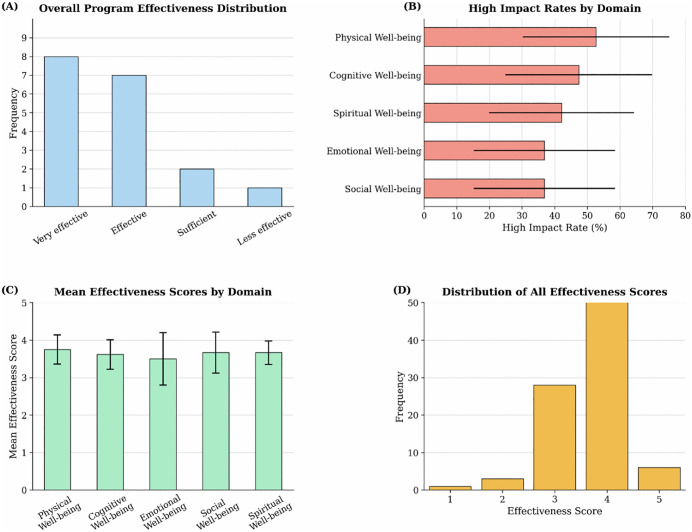
RT program effectiveness assessment showing (A) overall program effectiveness distribution, (B) high impact rates by well-being domain, (C) mean effectiveness scores by domain with confidence intervals, and (D) distribution of all effectiveness scores.

The RT program demonstrated varying levels of impact across different well-being domains (Table 3, Fig 2). Physical well-being showed the highest impact with a mean score of 3.75 ± 0.62, while emotional well-being had the lowest mean score of 3.50 ± 0.97. All domains achieved moderate to high impact ratings, with mean scores ranging from 3.50 to 3.75 on the 5-point scale.

**Table 3:** RT program impact ratings by well-being domain.

Well-being Domain	n	Mean ± SD	95% CI	High Impact Rate*	95% CI
Physical	19	3.75 ± 0.62	3.36-4.14	52.6%	30.2-75.1
Cognitive	19	3.62 ± 0.65	3.22-4.01	47.4%	24.9-69.8
Spiritual	19	3.67 ± 0.49	3.35-3.98	42.1%	19.9-64.3
Social	19	3.67 ± 0.71	3.12-4.21	36.8%	15.2-58.5
Emotional	19	3.50 ± 0.97	2.80-4.20	36.8%	15.2-58.5

*High Impact Rate = percentage rating “Very high impact” or “High impact”*

### Comparative analysis across domains

The overall effectiveness scores were not normally distributed (Shapiro-Wilk W = 0.589, p = 0.000), indicating the appropriateness of non-parametric statistical approaches. A Friedman test revealed no significant differences in perceived effectiveness across the five well-being domains (χ² = 0.563, p = 0.967), indicating relatively consistent impact perceptions across all areas. The domain effectiveness ranking by mean score was: Physical well-being (3.75)> Spiritual well-being (3.67) = Social well-being (3.67)> Cognitive well-being (3.62)> Emotional well-being (3.50). Effect size analysis between the highest (Physical) and lowest (Emotional) performing domains showed a small effect (Cohen’s d = 0.313), suggesting minimal practical differences in perceived effectiveness across domains.

### Organizational factors analysis

#### Program strengths and weaknesses.

**Fig 3:**
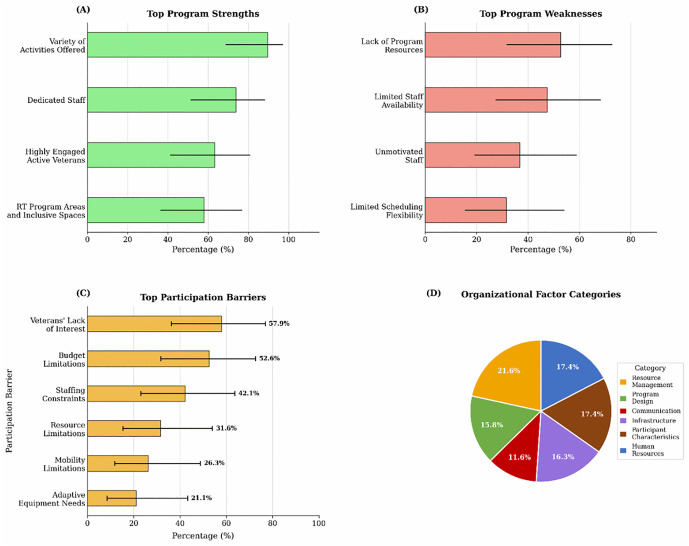
Organizational factors analysis showing (A) top program strengths, (B) primary program weaknesses, (C) participation barriers with confidence intervals, and (D) organizational factor categories distribution.

Analysis of organizational factors revealed distinct patterns in perceived program strengths and weaknesses (Table 4, Fig 3). Program strengths were dominated by activity variety (89.5%) and highly engaged active veterans (63.2%), while weaknesses centered on operational challenges including lack of program resources (52.6%) and limited staff availability (47.4%). A Mann-Whitney U test revealed significantly more strength selections per respondent (mean = 2.84) compared to weakness selections (mean = 2.11; U = 24.0, p = 0.010), indicating that leadership generally perceives more organizational assets than deficits.

**Table 4:** Organizational Strengths and Weaknesses.

Program Strengths	n	%	95% CI
Variety of activities offered	17	89.5	68.6-97.1
Highly engaged active veterans	12	63.2	41.0-80.9
Dedicated staff	14	73.7	51.2-88.2
RT program areas and inclusive spaces	11	57.9	36.3-76.9
**Program Weaknesses**			
Lack of program resources	10	52.6	31.7-72.7
Limited staff availability	9	47.4	27.3-68.3
Unmotivated staff	7	36.8	19.1-59.0
Limited scheduling flexibility	6	31.6	15.4-54.0
Low participation – less engaged veterans	5	26.3	11.8-48.8
Lack of variety in activities	3	15.8	5.5-37.6

### Participation factors and barriers

Leadership identified multiple factors affecting veteran participation, with staffing (47.4%) and communication (63.2%) being the most frequently cited (Table 5). Primary barriers to participation included veterans’ lack of interest (57.9%) and budget limitations (52.6%).

**Table 5:** Factors Affecting Participation and Primary Barriers.

Factors Affecting Participation	%	95% CI
Communication	63.2	41.0-80.9
Program content	52.6	31.7-72.7
Staffing	47.4	27.3-68.3
Accessibility	31.6	15.4-54.0
Scheduling	26.3	11.8-48.8
Available resources	31.6	15.4-54.0
Activity equipment	26.3	11.8-48.8
Encouragement to residents	52.6	31.7-72.7
**Primary Participation Barriers**		
Veterans’ lack of interest	57.9	36.3-76.9
Budget limitations	52.6	31.7-72.7
Staffing constraints	42.1	23.1-63.7
Resource limitations	31.6	15.4-54.0
Accessibility issues	15.8	5.5-37.6
Mobility limitations	26.3	11.8-48.8
Adaptive equipment needs	21.1	8.5-43.3

### Factor categorization and priority analysis

Organizational factors were systematically categorized into six domains to understand the breadth of organizational influences (Fig 3D). Resource Management emerged as the dominant concern, receiving the highest mention frequency (41 mentions, 21.6% of total responses), followed by Human Resources and Participant Characteristics (33 mentions each, 17.4%). The distribution across categories included Program Design (30 mentions, 15.8%), Infrastructure (31 mentions, 16.3%), and Communication (22 mentions, 11.6%). A chi-square test revealed no significant differences in category distribution (χ² = 5.916, p = 0.314, Cramér’s V = 0.079), indicating relatively balanced organizational concerns across all operational domains rather than concentration in a single area.

A comprehensive priority matrix was developed based on mention frequency and organizational impact to guide strategic decision-making (Table 6). Using frequency thresholds of ≥10 mentions for HIGH priority and 5–9 mentions for MEDIUM priority, eight factors emerged as requiring immediate attention.

**Table 6:** Organizational Priority Matrix by Strategic Importance.

Priority Level	Factor	Type	Mentions	%	Strategic Implication
**HIGH PRIORITY**					
1	Variety of activities offered	Strength	17	89.5	Leverage existing asset
2	Dedicated staff	Strength	14	73.7	Maintain/enhance capacity
3	Highly engaged active veterans	Strength	12	63.2	Expand engagement model
4	RT program areas and spaces	Strength	11	57.9	Infrastructure investment
5	Veterans’ lack of interest	Barrier	11	57.9	Critical intervention need
6	Lack of program resources	Weakness	10	52.6	Resource allocation priority
7	Budget limitations	Barrier	10	52.6	Financial planning focus
**MEDIUM PRIORITY**					
8	Limited staff availability	Weakness	9	47.4	Staffing strategy development

**Note:** Mentions = number of respondents selecting each factor (n = 19). Percentage = proportion of total respondents. Priority thresholds: HIGH = 10 or more mentions; MEDIUM = 5–9 mentions.

### Correlation analysis between organizational factors

Correlation analysis was conducted with recognition that results should be interpreted with appropriate caution given sample size constraints. Correlations with |r| > 0.3 were identified among organizational factors and are reported in S1 Table. All correlations were tested with Bonferroni correction applied across 35 comparisons, yielding an adjusted significance threshold of α = 0.0014. Three correlations survived Bonferroni correction and are considered statistically reliable: highly engaged active veterans and dedicated staff (r = 0.782, 95% CI [0.508, 0.912], t = 5.173, p < 0.001), RT program areas and dedicated staff (r = 0.701, 95% CI [0.362, 0.876], t = 4.053, p < 0.001), and limited staff availability with staffing constraints (r = 0.685, 95% CI [0.335, 0.869], t = 3.877, p = 0.001). Post-hoc power analysis confirmed strong statistical power for these three correlations at the unadjusted alpha (99.0%, 94.4%, and 92.8% respectively), with more modest power at the Bonferroni-corrected threshold (83.7%, 59.8%, and 54.9% respectively). Additional correlations exceeding the |r| > 0.3 threshold are reported in Table S1 for descriptive purposes only and should be treated as exploratory. Four descriptive patterns warranting further investigation were identified: strength synergies among organizational assets, barrier clustering indicating systemic capacity issues, strength-barrier tensions where organizational assets are strained by system limitations, and critical intervention points where resource investment may improve veteran engagement.

### Leadership involvement and organizational alignment

#### Mission-vision alignment analysis.

Leadership demonstrated strong confidence in the RT program’s alignment with organizational mission and vision (Table 7). The majority of respondents (76.5%) rated the program as highly aligned, with 35.3% indicating “completely aligned” and 41.2% “mostly aligned.” Only 5.9% perceived the program as “slightly aligned,” with no responses indicating poor alignment.

**Table 7:** Leadership Perspectives on Organizational Alignment and Strategic Importance.

RT Program Alignment with Mission/Vision	n	%	95% CI
Completely aligned	6	35.3	17.3-58.7
Mostly aligned	7	41.2	21.6-64.0
Moderately aligned	2	11.8	3.3-34.3
Slightly aligned	1	5.9	1.0-27.0
Not aligned	0	0.0	–
**Strategic Importance of Veterans’ Participation**			
Extremely important	10	58.8	35.4-82.2
Very important	6	35.3	12.6-58.0
Moderately important	1	5.9	0.0-17.1
**Importance of Increasing Participation for Mission Alignment**			
Extremely important	9	52.9	29.2-76.7
Very important	6	35.3	12.6-58.0
Moderately important	1	5.9	0.0-17.1

*High Alignment Rate (Completely + Mostly): 13/17 (76.5%, 95% CI: 56.3-96.6%) High Importance Rates: Participation (94.1%), Mission Alignment (88.2%)*

### Strategic importance assessment

Leadership unanimously recognized the strategic value of veteran participation, with 94.1% rating it as highly important (extremely or very important). Similarly, 88.2% considered increasing participation crucial for mission alignment. The correlation between current participation importance and increasing participation for mission alignment was moderate but non-significant (r = 0.211, p = 0.433), suggesting these are viewed as related but distinct strategic priorities.

### Leadership involvement patterns

Current leadership roles in RT program implementation showed varied engagement levels (Table 8). Strategic direction and support emerged as the primary leadership function (42.1%), followed by monitoring and reviewing progress (68.4%). Resource allocation received less emphasis (36.8%), while staff training represented the smallest leadership focus (52.6%).

**Table 8:** Leadership Involvement in RT Program Implementation.

Current Leadership Roles	n	%	95% CI
Monitoring and reviewing progress	13	68.4	46.0-84.6
Supporting staff training and development	10	52.6	31.7-72.7
Providing strategic direction and support	8	42.1	23.1-63.7
Allocation of additional resources and budget	7	36.8	19.1-59.0
**Desired Leadership Involvement Level**			
Moderately involved	6	35.3	12.6-58.0
Minimally involved	3	17.6	0.0-35.8

*Chi-square test for equal role distribution: χ² = 2.211, p = 0.530*

### Leadership experience and organizational perspective

Cross-tabulation analysis of the association between leadership experience and alignment perspectives involved a 4x4 contingency table with n = 17, resulting in expected cell frequencies of approximately 1.06, which seriously violated chi-square assumptions. A Monte Carlo permutation test (10,000 simulations) was therefore employed, revealing no significant association (p = 0.082). However, the large effect size (Cramér’s V = 0.613) suggests meaningful practical differences in how leaders with varying experience levels perceive organizational alignment, warranting cautious interpretation given the small sample size. Leaders with 1–5 years of experience showed the most diverse alignment perspectives, while those with 6–10 years demonstrated more consistent “mostly aligned” ratings. This pattern suggests that organizational perspective may stabilize with moderate experience but varies considerably among newer leadership personnel.

### Leadership-effectiveness relationship

Analysis of the relationship between leadership involvement and program effectiveness revealed a weak positive correlation (r = 0.335, p = 0.223, n = 15), indicating no significant association between current leadership engagement levels and perceived program effectiveness. However, effectiveness ratings varied meaningfully by mission-vision alignment levels: moderately aligned programs showed the highest effectiveness (M = 4.00 ± 0.00), followed by mostly aligned (M = 3.90 ± 0.22), completely aligned (M = 3.89 ± 0.11), and slightly aligned programs (M = 3.40 ± 0.00). This suggests that organizational alignment may be more predictive of effectiveness than leadership involvement intensity.

### Root cause analysis of participation barriers

Systematic analysis of low participation causes revealed distinct patterns in organizational versus individual factors (Table 9). Emotional and mood challenges emerged as the primary concern (68.4%), followed by physical limitations (63.2%) and social isolation (52.6%). Organizational factors, while present, received fewer mentions, with lack of interest in current activities (42.1%) and communication issues (21.1%) representing the primary institutional barriers.

**Table 9:** Root Cause Analysis of Low Participation by Priority Level.

Root Cause	Category	n	%	95% CI	Priority Level
Emotional and mood challenges	Individual	13	68.4	46.0-84.6	CRITICAL SEVERITY
Physical limitations of veterans	Individual	12	63.2	41.0-80.9	CRITICAL SEVERITY
Social isolation	Individual	10	52.6	31.7-72.7	CRITICAL SEVERITY
Lack of interest in current activities	Organizational	8	42.1	23.1-63.7	CRITICAL SEVERITY
Lack of communication/awareness	Organizational	4	21.1	8.5-43.3	Limited Control
Environmental challenges	Organizational	3	15.8	5.5-37.6	Limited Control

### Root cause categorization by organizational control

Root causes were categorized based on the degree of organizational control to distinguish between modifiable institutional factors and individual-level challenges. Of the total 50 mentions across all barriers, organizational factors accounted for 30% (15 mentions), while individual factors comprised 70% (35 mentions). This distribution highlights that the majority of participation barriers originate from individual-level limitations, such as emotional, physical, and social challenges, which may require therapeutic or personalized interventions beyond organizational adjustments.

### Resource allocation and strategic investment analysis

Comprehensive resource analysis revealed critical organizational capacity limitations (Table 10). The Resource Adequacy Index (RAI) was calculated as the ratio of resource adequacy mentions to total resource-related mentions across the survey: RAI = resource adequacy mentions / (resource adequacy mentions + resource deficit mentions) = 25 / 84 = 29.7%. Resource adequacy mentions comprised selections of dedicated staff (n = 14) and RT program areas and spaces (n = 11) as organizational strengths, while resource deficit mentions comprised all weakness and barrier selections related to staffing, funding, equipment, scheduling, and available resources (n = 59 total mentions). The overall RAI of 29.7% indicates insufficient organizational resources to support optimal program effectiveness. Resource-related factors dominated organizational concerns, accounting for 84.2% of barrier mentions, significantly exceeding strengths (57.9%), weaknesses (52.6%), and participation factors (31.6%). The resource allocation analysis indicates that Physical and Social well-being domains offer the highest return on investment potential, suggesting these areas should receive priority funding and resource allocation. The critical resource status (29.7% adequacy) provides quantitative justification for increased organizational investment in RT program infrastructure and operations.

**Table 10:** Resource allocation impact and investment recommendations.

Component	Current Status	Adequacy Level	Strategic Recommendation
**Resource Adequacy Index**	29.7%	Critical	Immediate investment required
**Resource Allocation Priority**			
Physical well-being programs	Score: 3.75	High ROI potential	Priority investment target
Social well-being programs	Score: 3.67	High ROI potential	Priority investment target
Cognitive well-being programs	Score: 3.62	Moderate ROI	Secondary investment
Emotional well-being programs	Score: 3.50	Moderate ROI	Secondary investment
Spiritual well-being programs	Score: 3.67	High ROI potential	Targeted enhancement

### Qualitative analysis of program effectiveness and organizational factors

#### Program impact narratives and organizational insights.

Analysis of 192 substantive qualitative responses (63.2% response rate) provided contextual validation of quantitative findings and revealed implementation insights not captured in structured questions. Seven distinct themes emerged, with Social Interaction (40 mentions) and Physical Health (36 mentions) showing the strongest prominence, directly supporting the quantitative effectiveness rankings (Table 11).

**Table 11:** Qualitative Theme Analysis and Quantitative Validation.

Qualitative Theme	Mentions	% of Total	Quantitative Alignment	Validation Status
Social Interaction	40	20.8%	Social well-being: 3.67 ± 0.71	**Strong Convergence**
Physical Health	36	18.8%	Physical well-being: 3.75 ± 0.62	**Strong Convergence**
Programming Quality	33	17.2%	Variety of activities: 89.5%	**Strong Convergence**
Emotional Well-being	28	14.6%	Emotional well-being: 3.50 ± 0.97	Moderate Convergence
Mental/Cognitive	23	12.0%	Cognitive well-being: 3.62 ± 0.65	Moderate Convergence
Engagement	22	11.5%	Highly engaged veterans: 63.2%	**Strong Convergence**
Staff/Resources	10	5.2%	Resource limitations: 52.6%	**Strong Convergence**
**Total**	**192**	**100.0%**		

### Key qualitative insights

#### Program effectiveness validation.

Leadership provided detailed impact descriptions that strongly supported quantitative domain rankings across all five well-being areas.

Physical well-being responses emphasized observable functional gains among residents. One respondent noted: *“mobility improvement, distraction from pain, strength and endurance, improved oxygenation, maintenance of current physical functioning levels,”* while another described how RT *“gives them more mobility, more confidence, able to do more for themselves.”* These narratives directly validate the highest quantitative effectiveness rating for physical well-being (3.75 ± 0.62).

Cognitive well-being responses highlighted memory stimulation and anxiety reduction as prominent benefits. One leader described residents’ engagement vividly: *“I’ve seen people’s faces light up when they have a memory of the good old days. While playing trivia it always reminds a resident of the past and they enjoyed telling everyone about it.”* Another noted *“improved executive functioning including memory, attention, concentration, decision-making, reasoning, and comprehension,”* supporting the moderate-high cognitive effectiveness rating (3.62 ± 0.65).

Emotional well-being narratives consistently described mood improvement and reduced loneliness. One respondent observed: *“We have had residents that have a death in the family or having a bad day but when they attend a group program they tend to cheer up.”* Another highlighted the breadth of emotional impact: *“increased socialization, reduced social isolation, collaboration, improved mood, joy, reminiscence, emotional expression, increased empathy.”* These narratives provide important context for the emotional well-being rating (3.50 ± 0.97), which showed the greatest variability across respondents.

Social well-being responses yielded some of the richest qualitative descriptions. One respondent described the transformative social impact: *“We’ve done many programs that involve the whole facility that have spawned many friendships, sparked interest in residents in activities that they might have been reluctant to try.”* Another captured the relational dimension of RT: *“social programs reduce isolation, make residents happy, joyful, and give meaning to their life.”* A third noted how RT supports residents’ transition into long-term care: *“improved social interactions, reduced isolation and helps to adjust to new life/environment from being a community member to being a long-term care resident.”*

Spiritual well-being responses emphasized peace, faith connection, and community among residents sharing common values. One leader stated: *“Recognizing each person’s spiritual interests is very important; it brings them peace and comfort,”* while another described broader benefits including *“mindfulness, keeping faith, peace of mind, connection with God.”*

### Critical organizational barriers revealed

Qualitative analysis identified specific implementation challenges not captured quantitatively.

**Staffing Practice Issues:** One respondent noted that *“staff are putting them to bed instead of bringing to programs,”* revealing operational barriers affecting participation despite program quality. A related concern highlighted systemic interdisciplinary gaps: *“lack of other discipline support for residents’ attendance and transport to programs,”* suggesting that RT program delivery depends on broader organizational cooperation beyond the RT team alone.

**Training Gaps:** Leadership responses revealed awareness deficits impacting implementation, with one respondent stating there is a *“lack of resources for staff to understand the value of Recreational Therapy.”* Another reinforced this concern: *“It would be really beneficial to offer further training to the RT team,”* pointing to a need for ongoing professional development.

**Program Continuity Concerns:** One respondent noted *“lots of changes to the entire program we once offered,”* indicating that organizational transitions may have disrupted established programming and contributed to reduced veteran engagement over time.

**Veteran-Specific Barriers:** One leader described generational preferences: *“We have a large amount of residents that are Vietnam Vets who prefer not to attend many programs. It is more of their preference to not attend big groups or be around people,”* identifying trauma-informed care needs requiring specialized approaches. Another respondent added that *“many residents prefer to spend leisure time in bed because of pain, or because they are more comfortable or tired,”* highlighting how individual health status intersects with program engagement.

### Innovation opportunities

Leadership suggested specific and actionable program enhancements. One respondent provided a comprehensive vision: *“I would like to see the team bridge the gap from offering daily activities to being more person-centered and objective programs to meet needs and goals of residents to live their highest quality of life through leisure. I would like to see the team be creative in programming to provide a resident with a sense of self by stepping out of the comfort zone and facilitating groups and activities that meet the recreation desires of residents.”* Additional suggestions included person-centered programming and community volunteers as resource enhancement strategies, alongside recommendations for *“workshops and functional activities that meet the interest of residents, expansion of current programs being offered, and not being afraid to try something new.”*

### Mixed-methods validation summary

The qualitative analysis provided convergent support for quantitative findings, with 6 of 7 major themes showing direct alignment with quantitative priority rankings. This pattern of convergence is consistent with but does not constitute formal triangulation in the strict methodological sense, as both data strands were derived from the same respondents within a single survey instrument. Rather, the integration serves to corroborate and contextualize quantitative findings through complementary qualitative perspectives, enhancing interpretive depth and credibility of the overall analysis [31]. The qualitative data particularly enhanced understanding of veteran-specific engagement challenges and operational barriers that were not fully captured in structured survey items, providing implementation nuances relevant for translating findings into actionable organizational improvements.

## Discussion

This study provides a comprehensive evaluation of the organizational factors that drive the effectiveness of RT programs in veterans’ nursing homes, and it highlights the critical role of leadership perspectives. The findings revealed that leadership generally perceived the RT program as highly effective across multiple well-being domains, particularly in supporting physical and social health. This is consistent with previous research, which has shown that RT contributes meaningfully to physical rehabilitation and enhances social interaction among older adults in long-term care settings [32,33]. However, although the program’s effectiveness was highly rated, several organizational challenges were evident, especially regarding resource limitations and staffing constraints. These challenges, combined with the Resource Adequacy Index of only 29.7%, indicate that the program is operating under severe capacity limitations, and this situation necessitates immediate strategic investment. While leadership emphasized the strengths of the RT program, such as the wide variety of activities offered and the dedication of the staff, these strengths alone cannot compensate for the structural weaknesses caused by insufficient resources. This finding agrees with previous studies that have emphasized how resource scarcity limits the breadth and quality of therapeutic programs and often restricts staff training and resident participation [34,35].

The correlation analysis demonstrated significant interdependences between organizational assets and participation barriers. For example, the strong positive association between dedicated staff and veteran engagement suggests that expanding staffing capacity may directly improve participation rates and program outcomes. Conversely, barriers such as limited staff availability and veterans’ lack of interest appeared to reinforce one another, which implies that staffing shortages not only hinder program delivery but may also contribute to declining veteran engagement over time.

Leadership expressed strong confidence in the alignment of the RT program with the facility’s mission and vision; however, the weak correlation between leadership involvement and program effectiveness suggests that involvement alone does not guarantee meaningful impact. Instead, more targeted leadership strategies and sustained engagement may be necessary to improve program outcomes. Additionally, differences in perceptions across leadership experience levels suggest that newer leaders may have more varied views on alignment and effectiveness, whereas more experienced leaders tend to hold more stable perspectives. This finding indicates that leadership tenure might influence organizational commitment and decision-making processes.

Furthermore, the root cause analysis offered valuable insights into the primary reasons for low participation. Emotional and mood challenges, along with physical limitations and social isolation, were identified as the most significant individual-level barriers. These results highlight that while addressing organizational constraints is important, tackling individual barriers through personalized, trauma-informed interventions is equally essential. Qualitative data revealed that generational preferences, particularly among Vietnam-era veterans, contribute to lower engagement in group activities, with respondents noting that many Vietnam-era residents prefer not to attend group programs and instead favor solitary or small-group interactions. This pattern offers a direct explanatory mechanism for the emotional well-being domain’s comparatively lower effectiveness rating (3.50 ± 0.97) relative to other domains. Specifically, RT programs that rely predominantly on group-based formats may systematically underserve veterans whose trauma histories and generational norms around emotional expression discourage participation in communal settings, thereby limiting the emotional therapeutic benefit they receive. Therefore, individualized programming that accounts for trauma histories and personal preferences, including one-on-one visits, small-group alternatives, and person-centered activity planning, may be necessary to improve emotional well-being outcomes among this population.

The qualitative findings also uncovered operational issues that were not fully captured in the quantitative results. Leadership described staffing practices where staff prioritized other tasks over facilitating recreational therapy, which inadvertently limited veteran participation. Additionally, leaders noted a lack of staff understanding regarding the therapeutic value of recreational therapy, which suggests that improving education and raising awareness among care staff are vital steps toward creating a more supportive organizational culture. The alignment between quantitative priorities and qualitative themes validated the results, and it uncovered practical challenges that would not have been evident through quantitative analysis alone. Leadership recommendations, such as expanding person-centered programming, introducing workshops that reflect resident interests, and increasing the involvement of community volunteers, offer realistic and actionable strategies for overcoming the identified barriers.

### Implications for practice and policy

These findings have several important implications for recreational therapy practice in veterans’ nursing homes. First, the organizational priority matrix provides an evidence-based framework for strategic planning that balances leveraging existing strengths (activity variety, dedicated staff) with addressing critical deficiencies (resource limitations, staffing constraints). Healthcare administrators can use this framework to justify resource requests and prioritize improvement initiatives based on empirical evidence rather than assumptions. Second, the identification of veteran-specific factors requiring specialized approaches suggests that standard recreational therapy models may require adaptation for military populations. The need for trauma-informed programming and generational considerations indicates that staff training and program development should incorporate military cultural competency and trauma-sensitive practices. This finding supports policy recommendations for specialized training requirements in veterans’ care facilities. Third, the critical resource adequacy finding (29.7%) provides quantitative justification for increased organizational investment in recreational therapy infrastructure and operations. Policy makers can use these data to support funding decisions and quality standards that ensure adequate resource allocation for program effectiveness and sustainability.

### Study Limitations and Future Research

Several limitations should be considered when interpreting these findings. The single-site design limits generalizability to other veterans’ nursing homes, and the small sample size (n = 19) may affect statistical power for detecting smaller effect sizes [36,37]. Post-hoc power analysis revealed substantial variability across key statistical tests. The correlation between engaged veterans and dedicated staff (r = 0.782) achieved excellent power (99%), supporting confidence in this finding. However, the Friedman test comparing effectiveness across well-being domains yielded a negligible effect size (Kendall’s W = 0.007) with post-hoc power of approximately 8%, indicating that the non-significant result should be interpreted with caution rather than as definitive evidence of domain equivalence. Similarly, the correlation between leadership involvement and program effectiveness (r = 0.335, n = 15) achieved only 24% power, suggesting that a meaningful relationship may exist but remained undetected at this sample size; achieving 80% power for this effect size would require approximately 67 participants.

The sample composition, with 68.8% front-line staff and limited senior leadership representation, may introduce response bias toward operational rather than strategic perspectives on program effectiveness. Additionally, voluntary participation and variable completion rates across survey sections (59.6% to 100%) suggest potential non-response bias, particularly for organizational factors questions where completion was lowest. Cross-sectional design prevents causal inferences about relationships between organizational factors and program effectiveness [38]. Future research should employ multi-site designs to examine organizational factor patterns across different veterans’ care environments and investigate the relationship between leadership perspectives and objective program outcomes. Longitudinal studies could examine how organizational changes influence program effectiveness over time [39,40], while mixed methods approaches incorporating resident and clinical staff perspectives could provide more comprehensive understanding of program impacts and implementation dynamics.

## Conclusion

This mixed-methods study provides the first comprehensive examination of organizational factors influencing recreational therapy program effectiveness in veterans’ nursing homes from leadership perspectives. The research demonstrates that RT programs achieve strong effectiveness across multiple well-being domains (78.9% highly effective), yet critical resource constraints (29.7% adequacy index) threaten program sustainability. The study identified actionable organizational priorities through an evidence-based matrix framework and revealed veteran-specific implementation challenges requiring trauma-informed approaches. The strong convergence between quantitative and qualitative findings validates mixed-methods approaches in healthcare intervention research, while identifying Physical and Social well-being domains as high return-on-investment targets for resource allocation. To translate these findings into practice, healthcare administrators should consider specific staffing strategies including cross-training ancillary staff to support RT program delivery during periods of high demand, implementing flexible scheduling models to improve coverage across shifts, and establishing interdisciplinary collaboration protocols to ensure that all care staff actively support resident transport and attendance at RT programs. Addressing staffing constraints through these targeted approaches may directly improve veteran engagement, given the strong positive correlation identified between dedicated staff and active veteran participation (r = 0.782). With respect to individualized programming, findings indicate that standard group-based RT models may be insufficient for veterans with trauma histories or generational preferences for non-group participation, particularly among Vietnam-era veterans. Facilities should therefore develop person-centered programming frameworks that incorporate trauma-informed principles, offer one-on-one and small-group alternatives alongside large group activities, and systematically assess individual resident leisure preferences to guide program design. Community volunteer integration represents an additional low-cost strategy for expanding programming capacity while maintaining individualized engagement.

These findings contribute to implementation science literature and generate immediately applicable tools including the organizational priority matrix and resource allocation framework that healthcare administrators and policymakers can implement to enhance program effectiveness. Consequently, this work provides an evidence-based foundation for quality improvement initiatives and advances the field of recreational therapy while improving care quality for the aging veteran population.
